# Evolution of pathogenicity traits in the apple scab fungal pathogen in response to the domestication of its host

**DOI:** 10.1111/j.1752-4571.2012.00246.x

**Published:** 2012-11

**Authors:** Amandine Lê Van, Pierre Gladieux, Christophe Lemaire, Amandine Cornille, Tatiana Giraud, Charles-Eric Durel, Valérie Caffier, Bruno Le Cam

**Affiliations:** 1 INRA, UMR1345, IRHS (INRA, Agrocampus-Ouest, Université d’Angers)SFR QUASAV, Beaucouzé, France; 2 CNRS, UMR 8079, Ecologie, Systématique et Evolution, Univ. Paris-SudOrsay, France; 3 AgroParisTechOrsay, France; 4 Université d’Angers, UMR1345 IRHS (INRA, Agrocampus-Ouest, Université d’Angers)SFR QUASAV, Angers, France

**Keywords:** apple scab, coevolution, disease emergence, plant–microbe interactions, wild crop relatives

## Abstract

Understanding how pathogens emerge is essential to bring disease-causing agents under durable human control. Here, we used cross-pathogenicity tests to investigate the changes in life-history traits of the fungal pathogen *Venturia inaequalis* associated with host-tracking during the domestication of apple and subsequent host-range expansion on the wild European crabapple (*Malus sylvestris*). Pathogenicity of 40 isolates collected in wild and domesticated ecosystems was assessed on the domesticated apple, its Central Asian main progenitor (*M*. *sieversii*) and *M. sylvestris*. Isolates from wild habitats in the centre of origin of the crop were not pathogenic on the domesticated apple and less aggressive than other isolates on their host of origin. Isolates from the agro-ecosystem in Central Asia infected a higher proportion of plants with higher aggressiveness, on both the domesticated host and its progenitor. Isolates from the European crabapple were still able to cause disease on other species but were less aggressive and less frequently virulent on these hosts than their endemic populations. Our results suggest that the domestication of apple was associated with the acquisition of virulence in the pathogen following host-tracking. The spread of the disease in the agro-ecosystem would also have been accompanied by an increase in overall pathogenicity.

## Introduction

The domestication of plants, expanding global trade and agriculture are major drivers of plant pathogen emergence (Pysek et al. 2010). Understanding how pathogens emerge on domesticated plants and spread in the agro-ecosystem is essential to bring disease-causing agents under durable human control (Anderson et al. 2004). Population genetic analyses on samples from domesticated and wild hosts have provided important insights into the origins of fungal pathogens (Gomez-Alpizar et al. 2007; Frenkel et al. 2010; Stukenbrock et al. 2011; Torriani et al. 2011). The emergence of pathogens on domesticated hosts can result from the colonization of a novel host or from a process of host-tracking where the pathogen simply follows its current host along the domestication process (e.g. Munkacsi et al. 2008; Gladieux et al. 2010; Giraud et al. 2010; Hansen 1987; Stukenbrock and McDonald 2008). Colonization of a novel host can be due to host-range expansion (i.e. colonization of a new host species while remaining pathogenic on the ancestral host) or host-shift (i.e. colonization of a new host species associated with the loss of the ability to infect the ancestral host). By definition, host-shifts involve very strong (qualitative) host specialization and can lead to rapid speciation through the evolution of an association between genes involved in host adaptation and genes conferring reproductive isolation (Couch et al. 2005; Giraud et al. 2010). In the case of host-range expansion or host-tracking, specialization is not an obligate outcome but quantitative specialization to the new host can nonetheless be observed, with a lower performance on the ancestral host of pathogen populations from the new host compared to native populations (Fry 2003; Sicard et al. 2007). In fungal plant pathogens, specialization is favoured by some particular life-history traits including a large number of spores, mating within the host and high selection coefficients on a limited number of genes (Giraud et al. 2010).

In addition to the divergent selective pressures caused by the genetic differences among ancestral and domesticated hosts, pathogens are also exposed to a novel habitat, the agro-ecosystem, drastically different from natural ecosystems. The particular features of the human-engineered environment are thought to further enhance pathogen specialization to the domesticated hosts (Stukenbrock and McDonald 2008). Unlike natural plant populations, the high density and genetic uniformity of cultivated plant populations are highly conducive to pathogen transmission between infected and noninfected hosts, which favours more aggressive pathogens (Anderson and May 1982; Hochberg 2000; Thrall et al. 2007). Moreover, cultivated crops represent large targets for pathogens shifting from other hosts, while the large and widely connected pathogen populations associated with the agro-ecosystem are potential reservoirs for novel epidemics on naïve hosts that do not have evolved defence mechanisms (Couch et al. 2005). The vast-scale and homogenous availability of nutrients in the agro-ecosystem is also expected to enable the development of very large populations of pathogens, thereby increasing the efficiency of selection and accelerating adaptation (Karasov et al. 2010).

In analogy with the common suite of morphological and physiological traits that distinguish crops from their wild ancestors (Doebley et al. 2006; Zeder et al. 2006), the changes in life-history traits of pathogens adapting to domesticated hosts and to the agro-ecosystem can be regarded as a ‘domestication syndrome’. Unlike plants, however, for which the domestication syndrome has been extensively investigated, the study of the evolutionary changes in pathogens associated with the domestication of plants and ecosystems is still in its infancy. This might be related to the lack of archaeological data on pathogens and to the difficulty in identifying, getting access and collecting samples in the centre of origins of diseases. Studies on the rice blast pathogen, *Magnaporthe oryzae*, have demonstrated differences in pathogenicity traits between populations infecting domesticated rice and the ancestral host of the pathogen, Setaria millet. Isolates from Setaria millet were either not virulent on rice or less aggressive than isolates from rice (Couch et al. 2005). Similarly, the pathogen *Rhynchosporium* shifted from an unidentified ancestor to cultivated barley and rye and speciated after adaptation to its new hosts at the time of domestication of cereals in the Fertile Crescent (Zaffarano et al. 2008).

The pathosystem *Malus* spp.-*Venturia inaequalis* (apple scab) is an excellent system to investigate the changes in life-history traits of pathogens adapting to domesticated hosts and to the agro-ecosystem. First, the life-history traits of *V. inaequalis* confer to this ascomycete an important evolutionary potential (McDonald and Linde 2002). The fungus reproduces both asexually during spring and summer (epidemic phase) and sexually during winter (saprotrophic phase). Reproduction occurs between strains of opposite mating types that have infected the same leaf. This reproductive system, where mating occurs between individuals that were able to grow on the same host genotype, is highly conducive to rapid ecological divergence (Giraud et al. 2010; Gladieux et al. 2011). Second, the story of the apple domestication is well documented. Historical information (Juniper and Mabberley 2006) and (partial) genetic evidence (Harris et al. 2002; Velasco et al. 2010) suggested that the centre of origin of the cultivated apple (*M.* × *domestica*) was Central Asia, where the wild apple *M. sieversii*, its main progenitor, forms forests (Harris et al. 2002). From Central Asia, the domesticated apple was moved westward to Europe and eastward to China following the Silk Road (Juniper and Mabberley 2006). During the spread of apple cultivation, several other *Malus* species may have contributed to the gene pool of *M.* × *domestica*. While the domesticated varieties appear closely related to *M. sieversii* (Velasco et al. 2010), a possible contribution from the European crabapple *M. sylvestris* to the domesticated apple gene pool is still debated (Coart et al. 2006; Harrison and Harrison 2011; Micheletti et al. 2011; A Cornille, P. Gladieux, I. Roldán-Ruiz, F. Laurens, B. Le Cam, M. J. M. Smulders, A. Nersesyan, J. Clavel, M. Olonova, L. Feugey, I. Gabrielyan, X. G. Zhang, M. I. Tenaillon, T. Giraud, unpublished manuscript). The speciation between European and Central Asian wild apples likely occurred during Pleistocene repeated glaciations owing to the retreat and fragmentation of an ancient corridor of Tertiary temperate forests that ranged from the Atlantic Ocean to Bering (Juniper and Mabberley 2006). *Malus sylvestris* is now considered as an endangered tree species in some European regions, with a very scattered distribution (Stephan et al. 2003; A Cornille, P. Gladieux, I. Roldán-Ruiz, F. Laurens, B. Le Cam, M. J. M. Smulders, A. Nersesyan, J. Clavel, M. Olonova, L. Feugey, I. Gabrielyan, X. G. Zhang, M. I. Tenaillon, T. Giraud, unpublished manuscript). Third, population genetics studies on *V. inaequalis* provided important clues about the evolutionary history of this pathogen. A previous study on the population structure of *V. inaequalis* showed that the pathogen shared a common origin with its host in Central Asia (Gladieux et al. 2008). A subsequent study on populations of *V. inaequalis* infecting the wild apples *M. sieversii* and *M. sylvestris* pinpointed *M. sieversii* as the host of origin of the fungus (Gladieux et al. 2010). Results were consistent with a host-tracking scenario in which *V.* *inaequalis* spread into Europe together with the domesticated apple and subsequently expanded its range to *M. sylvestris*, previously free of apple scab. Population genetic analyses indicated that apple domestication had a strong impact on the population structure of the pathogen: apple domestication was associated with significant changes in the genetic differentiation of *V. inaequalis* populations in their centre of origin but had little impact on historical demography and mating system of the fungus (Gladieux et al. 2010). Three distinct gene pools were indeed identified based on microsatellite data by Gladieux et al. (2010): a population geographically restricted to the south-eastern mountains of Kazakhstan parasitizing *M. sieversii* (CAM population), an Asian population infecting *M.* × *domestica* and *M. sieversii* in peri-urban or agricultural areas (CAP population) and a European population present on *M.* × *domestica* and *M. sylvestris* (EU population) (Gladieux et al. 2010). Gladieux et al. (2010) hypothesized that the mountain population (CAM) could represent a relict of the ancestral populations that infected *M. sieversii* before apple domestication and from which the other populations would have diverged following domestication. The CAM population would represent an undisturbed population of the pathogen from natural ecosystems, while the CAP and EU populations would be evolved populations in contact with the agro-ecosystem.

Here, we used cross-inoculation tests to investigate the changes in pathogenicity traits of the apple scab fungus *V. inaequalis* associated with the domestication and spread of its host. Pathogenicity of 40 isolates collected in wild and domesticated ecosystems was assessed on the domesticated apple (*Malus* × *domestica*), its Central Asian main progenitor (*M*. *sieversii*) and the wild European crabapple (*M*. *sylvestris*). Two components of pathogenicity were analysed: virulence, that is, the ability to infect a given host genotype, and aggressiveness, that is, the severity of the disease in successful infections. We tested the hypotheses that agro-ecosystem features such as high host density favoured pathogen specialization on *M*. × *domestica* as well as an increase in aggressiveness, while the features of the European forest ecosystem with a very scattered host distribution did not lead to the specialization on *M. sylvestris*. We tested more specifically the following hypotheses: (i) host-tracking of *V. inaequalis* from the wild ancestor to the cultivated apple has been associated with a gain in virulence; evidence would be that isolates from the wild Asian progenitor are unable to cause disease on *M.* × *domestica*; (ii) adaptation to the cultivated apple has been associated with increased overall pathogenicity; evidence would be that isolates from domesticated apple trees are more aggressive or more frequently virulent on the wild Asian progenitor than the endemic isolates; (iii) the emergence of apple scab on wild crabapple populations in Europe was due to a host-range expansion and not a host-shift of *V. inaequalis* populations from agro-ecosystems; evidence would be that isolates from crabapple trees are still able to cause disease on the domesticated trees; iv) the host-range expansion on *M. sylvestris*, nevertheless, resulted in a certain degree of specialization; evidence would be that crabapple isolates show lower aggressiveness or lower frequency of virulence on domesticated trees than isolates from *M.* × *domestica*.

## Materials and methods

### Fungal isolates

This study was based on a total of 40 isolates of *V. inaequalis* (Table 1) sampled on *M. sieversii*, *M.* × *domestica* and *M. sylvestris.* Three core collections of *V. inaequalis* were previously constructed, one per *Malus* species of origin, as maximizing neutral genetic diversity among isolates genotyped with 12 SSR loci (Lê Van et al. 2011). Each core collection was constituted by 15 isolates except the ‘*M*. × *domestica* core collection’ constituted by 10 isolates. The isolates originating from CAM, CAP or EU populations (Gladieux et al. 2010) were classified into five pools labelled after their geographic origin (Asia or Europe), the environment of origin (wild or agro-ecosystem) and the host of origin (*M. sieversii*, *M*. × *domestica* or *M. sylvestris*): ‘WildAsiaSiev’, ‘AgroAsiaSiev’, ‘AgroAsiaDom’, ‘AgroEuDom’ and ‘WildEuSylv’ (Table 1).

**Table 1:** Description of the *Venturia inaequalis* isolates used in this study

Isolate	Country of origin	Sampled year	Population name	*Malus* host (Cultivar)
2217	Kazakhstan	2006	WildAsiaSiev†	*M. sieversii*
2219	Kazakhstan	2006	WildAsiaSiev†	*M. sieversii*
2220	Kazakhstan	2006	WildAsiaSiev†	*M. sieversii*
2221	Kazakhstan	2006	WildAsiaSiev†	*M. sieversii*
2222	Kazakhstan	2006	WildAsiaSiev†	*M. sieversii*
2223	Kazakhstan	2006	WildAsiaSiev†	*M. sieversii*
2224	Kazakhstan	2006	WildAsiaSiev†	*M. sieversii*
2225	Kazakhstan	2006	WildAsiaSiev†	*M. sieversii*
2227	Kazakhstan	2006	AgroAsiaSiev‡	*M. sieversii*
2228	Kazakhstan	2006	AgroAsiaSiev‡	*M. sieversii*
2229	Kazakhstan	2006	AgroAsiaSiev‡	*M. sieversii*
2230	Kazakhstan	2006	AgroAsiaSiev‡	*M. sieversii*
2231	Kazakhstan	2006	AgroAsiaSiev‡	*M. sieversii*
2233	China	2005	AgroAsiaSiev‡	*M. sieversii*
2234	China	2005	AgroAsiaSiev‡	*M. sieversii*
2278	China	2005	AgroAsiaDom‡	*M.* × *domestica* (Golden Delicious)
2279	China	2005	AgroAsiaDom‡	*M.* × *domestica* (Gala)
2281	China	2005	AgroAsiaDom‡	*M.* × *domestica* (New Century)
2284*	China	2005	AgroAsiaDom‡	*M.* × *domestica* (New Century)
2285	China	2005	AgroAsiaDom‡	*M.* × *domestica* (Gala)
2286	France	2005	AgroEuDom§	*M.* × *domestica* (Mutsu)
2288	France	2005	AgroEuDom§	*M.* × *domestica* (Mutsu)
2289	France	2005	AgroEuDom§	*M.* × *domestica* (Mutsu)
2291	Spain	2005	AgroEuDom§	*M.* × *domestica* (Wellspur)
EU-D-16	Germany	1999	AgroEuDom§	*M.* × *domestica* (Coop 9)
2237	France	2005	WildEuSylv§	*M. sylvestris*
2238*	France	2005	WildEuSylv§	*M. sylvestris*
2239*	France	2005	WildEuSylv§	*M. sylvestris*
2240*	France	2005	WildEuSylv§	*M. sylvestris*
2241*	France	2005	WildEuSylv§	*M. sylvestris*
2245	France	2005	WildEuSylv§	*M. sylvestris*
2246*	France	2005	WildEuSylv§	*M. sylvestris*
2247	France	2005	WildEuSylv§	*M. sylvestris*
2248*	France	2005	WildEuSylv§	*M. sylvestris*
2249*	France	2005	WildEuSylv§	*M. sylvestris*
2251*	France	2005	WildEuSylv§	*M. sylvestris*
2252*	France	2005	WildEuSylv§	*M. sylvestris*
2254*	France	2005	WildEuSylv§	*M. sylvestris*
2255*	France	2005	WildEuSylv§	*M. sylvestris*
2256	France	2005	WildEuSylv§	*M. sylvestris*

* These isolates were inoculated onto *M.* × *domestica* and *M. sylvestris* but not onto *M. sieversii*.

† This population belongs to the previously identified CAM (Central Asian Mountains) population (Gladieux et al. 2010).

‡ This population belongs to the previously identified CAP (Central Asian Plains) population.

§ This population belongs to the previously identified EU (European) population.

### Plant material

Two cultivars of *M*. × *domestica*, three accessions of *M. sieversii* (GMAL 3619.b, PI 633797.d and PI 633799.e) and four accessions of *M. sylvestris* (X 9650, X 9651, X 9653 and X 9654) were used in this study. The two cultivars, Gala and Top Red Delicious (latter on called ‘Top Red’), are extensively cultivated worldwide. *Malus sieversii* accessions were collected in Kazakhstan in two different localities (Figure S1). GMAL 3619.b and PI 633799.e were collected from the Tarbagatai mountain range, and PI 633797.d was collected from the Djungarsky mountain range (Forsline et al. 2010; USDA website: http://www.ars-grin.gov/cgi-bin/npgs/html/search.pl). *Malus sylvestris* accessions were collected in the French forest of Rambouillet. Budwoods were grafted on ‘MM106’ apple rootstocks and then maintained in a greenhouse. The plants of *M. sylvestris* used in pathogenicity tests were genotyped using microsatellite markers, and their assignment to the gene pool of their putative species of origin was checked using a reference data set (Data S1).

### Cross-pathogenicity tests

The 40 isolates were inoculated onto all host genotypes during three rounds of experiments per host species. For each experiment, one to six isolates of each fungal population were inoculated onto one *Malus* species. For inoculations onto *M. sieversii*, 28 of 40 isolates were inoculated because of limited plant material available (Table 1). Only actively growing plants with uniform growth were chosen and transferred to a quarantine-controlled climate chamber for subsequent inoculations. We used a quarantine room because of the exotic origin of numerous isolates whose unknown virulence may present a risk. Inocula were obtained by growing monoconidial isolates of the fungus on cellophane sheets deposited onto malt agar medium (Bus et al. 2005). Monoconidial suspensions of each isolate were adjusted to a concentration of 1.5 × 10^5^ conidia/mL. Germination rates were assessed for each monoconidial suspension on malt agar plates to check for the viability of conidia. Germination rates ranged from 34% to 95% depending on the isolate and the experiment, with more than 64% reached in 75% of cases. Each isolate was sprayed using an air pressure hand-sprayer on four to five replicates of each host genotype. All leaves were inoculated. For the first 48 h after inoculation, the plants were kept in darkness with humidity maintained at 100% and temperature at 18°C to allow conidia germination and fungal infection. Humidity was then reduced to 80% with 16-h light per day. Percentage of each leaf showing sporulation was scored visually at 14, 21 and 28 days after inoculation (dai) on an ordinal scale, ranging from 0 (no sporulation) to 8 (100% of leaf area showing sporulation) (Lê Van et al. 2011). For virulence, each replicate was either scored as infected or not (no visible sporulating symptoms at 28 days).

### Data analyses

#### Analyses of virulence

Virulence was defined as the ability of an isolate to produce sporulating symptoms on a host genotype. A Pearson’s chi-squared test was performed on contingency tables to test for independence between virulence and population of origin. Because of expected cell count below five, *P*-values were computed for a Monte Carlo test (Hope 1968) with 1 × 10^5^ replicates. When virulence and the population of origin were not independent, multiple comparisons were made using Pearson’s chi-squared tests on two-by-two contingency tables using the Bonferroni correction. A Pearson’s chi-squared test was also conducted to test for independence between virulence and *Malus* species tested.

#### Analyses of aggressiveness

Aggressiveness was measured as the area under the disease progress curve (AUDPC) calculated on the sporulation percentage of the most diseased leaf of each replicate. The ‘AUDPC’ variable was analysed using a linear mixed-effect model (LME). The ‘isolate’ was treated as random factor and nested in the population of origin. The cultivars were treated as fixed factors. A variance function was used for modelling the within-group heteroscedasticity. Each factor (isolate, population of origin, *Malus* species of origin, tested cultivar and round of experiment) was included in the model based on an ascendant selection using BIC (Bayesian Information Criterion) to select the best model (Pinheiro and Bates 2000). The model was fitted by maximizing the log-likelihood. All statistical analyses (virulence and aggressiveness) were performed using the ‘nlme’ package (Pinheiro et al., 2008) in R version 2.10.1 (R-Development-Core-team, 2008).

## Results

### Virulence

**Figure 1:**
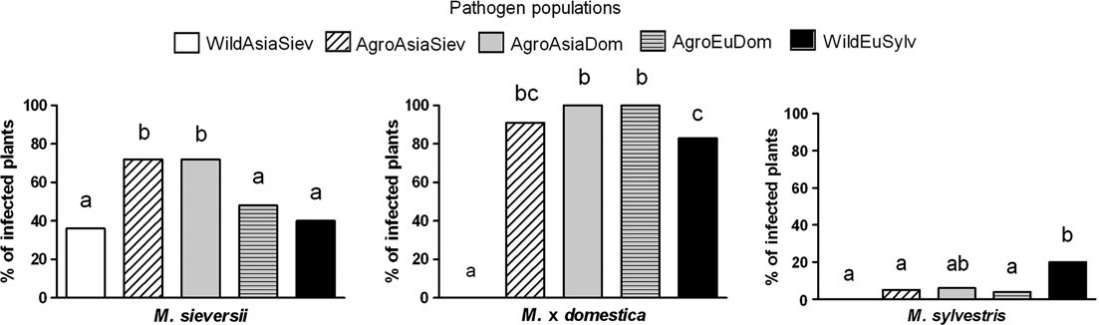
Percentage of plants of three *Malus* species infected by five *Venturia inaequalis* populations. Note that an empty place indicates zero infected plant for the corresponding pathogen population. Virulence was measured on three accessions for *M. sieversii*, two cultivars for *M*. × *domestica* and four accessions for *M. sylvestris*. Different letters indicate significant differences between populations (*P* < 0.05) (Pearson’s chi-squared tests with Bonferroni correction on two-by-two contingency tables).

*Malus sieversii* plants were significantly more often infected by isolates from Central Asia sampled in the agro-ecosystem area, either from *M. sieversii* (AgroAsiaSiev population) or from *M*. × *domestica* (AgroAsiaDom population), than by isolates sampled in the wild area in Central Asia (WildAsiaSiev population) or isolates from Europe, either from wild or from agro-ecosystem (Pearson’s chi-squared tests on two-by-two contingency tables; Fig. 1). Isolates belonging to the Asian population from the agro-ecosystem were able to infect up to three *M. sieversii* accessions, whereas isolates from Europe were able to infect up to two accessions. Only one of the three accessions was infected by isolates from the WildAsiaSiev population (Table 2). The higher frequency of virulence on *M. sieversii* in fungal populations from Asian agro-ecosystems suggests that the spread of the disease on the domesticated apple has been associated with an increase in pathogenicity of *V. inaequalis*.

**Table 2:** Number of virulent isolates of *Venturia inaequalis* out of the number of isolates tested for each genotype of three *Malus* species

*M. sieversii* accessions				*M.* × *domestica* cultivars		*M. sylvestris* accessions			
		
Pathogen population	GMAL 3619.b	PI 633 797.d	PI 633 799.e	Gala	Top Red	X9650	X9651	X9653	X9654
WildAsiaSiev	8/8	0/8	0/7	0/8	0/7	0/8	0/8	0/8	0/8
AgroAsiaSiev	7/7	3/7	5/7	6/7	7/7	0/7	0/7	2/7	0/6
AgroAsiaDom	4/4	3/4	3/4	5/5	5/5	0/5	0/5	1/4	0/4
AgroEuDom	5/5	1/5	1/5	5/5	5/5	0/5	0/5	0/5	1/5
WildEuSylv	4/4	0/4	1/4	13/15	12/15	1/14	2/15	4/12	6/14

None of the *M*. × *domestica* cultivars was infected by the WildAsiaSiev population. *Malus* × *domestica* cultivars were significantly less frequently infected by the WildAsiaSiev population than by other pathogen populations (Pearson’s chi-squared tests on two-by-two contingency tables; Fig. 1). All other populations were able to infect both *M* × *domestica* cultivars (Fig. 1). The lack of pathogenicity of isolates from wild habitats of the centre of origin of the crop when inoculated on the domesticated apple indicates that the host-tracking of *V. inaequalis* from the wild ancestor to the cultivated apple has demanded acquisition of new virulence.

Although isolates from both *M. × domestica* and *M. sylvestris* were able to infect *M.* × *domestica* cultivars, the number of infected plants was significantly lower when challenged with isolates from *M. sylvestris* (WildEuSylv population) than with isolates from *M.* × *domestica* either collected in Asia or in Europe (χ^2^ = 9.44; *P* < 0.01 and χ^2^ = 6.82; *P* < 0.01, respectively). This suggests a certain degree of specialization by isolates parasitizing the European crabapple. No significant differences were observed between isolates from *M*. × *domestica* from either Asia or Europe (*P* = 1) (Fig. 1).

*Malus sylvestris* plants were significantly more frequently diseased after inoculation by isolates from *M. sylvestris* than by isolates from other *Malus* species (Pearson’s chi-squared tests on two-by-two contingency tables; Fig. 1), except for AgroAsiaDom. Moreover, some isolates from *M*. *sylvestris* were able to infect up to three different *M. sylvestris* accessions, whereas isolates from other populations were able to infect a single accession. This suggests that the colonization of *M. sylvestris* has demanded a certain degree of adaptation.

The number of diseased plants was not significantly different when challenged with isolates from the agro-ecosystem in Asia sampled on *M*. × *domestica* or on *M*. *sieversii* (χ^2^ = 0.03; *P* = 1). Similar to the pattern of virulence observed on *M.* × *domestica*, the eight isolates from WildAsiaSiev were not able to infect *M. sylvestris* accessions (Fig. 1). *Malus sylvestris* accessions were resistant to a significantly higher number of isolates than *M.* × *domestica* or *M. sieversii* accessions (χ^2^ = 356.28; *P* < 0.0001).

### Aggressiveness

Data of all experiments conducted on the same *Malus* species were pooled. The experiment factor had no significant effect and did not improve LME models. As a consequence, it was not eventually included in the models. The within-group heteroscedasticity was modelled as a power function of mean fitted values.

**Figure 2:**
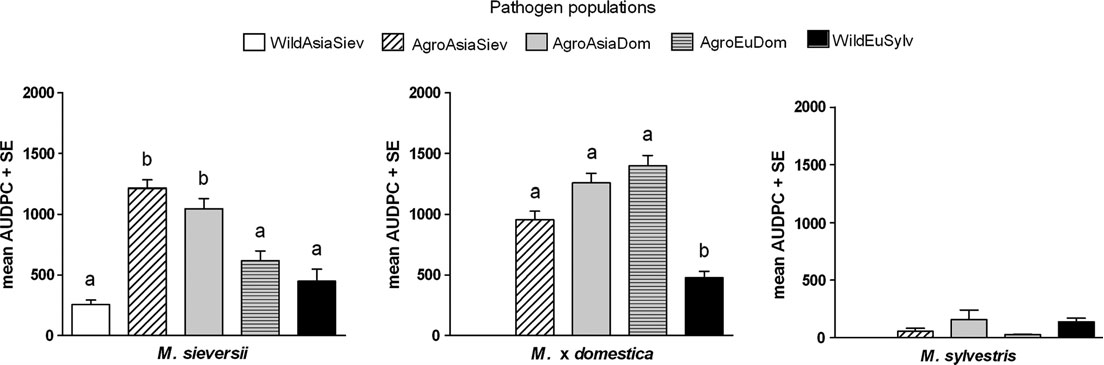
Mean area under the disease progress curve (AUDPC) (+SE) of five *Venturia inaequalis* populations inoculated onto three *Malus* species. AUDPC was measured on one accession for *Malus sieversii*, two cultivars for *M*. × *domestica* and two accessions for *M. sylvestris*. Different letters indicate significant differences between populations (*P* < 0.05), parameters being estimated by the maximum-likelihood algorithm in the linear mixed-effect model.

Because a single *M. sieversii* accession was susceptible to all pathogen populations, statistical analyses were conducted only for this accession (GMAL 3619.b). The factor ‘population of origin’ explained the area under the disease progress curve (AUDPC) variance (BIC = 3158) and significantly improved the null model (BIC = 3176; *P* < 0.0001). Isolates from Asia collected in the agro-ecosystem, either from *M. sieversii* or from *M*. × *domestica*, were significantly more aggressive than isolates from other populations on the *M. sieversii* accession (Fig. 2). The AgroAsiaSiev and the AgroAsiaDom populations were on average fivefold and fourfold, respectively, more aggressive than the WildAsiaSiev population. This higher aggressiveness of populations from agro-ecosystems on *M. sieversii* is another evidence indicating an increase in overall pathogenicity following the spread of the pathogen on the domesticated apple.

Aggressiveness of isolates from WildAsiaSiev was significantly lower than aggressiveness of isolates from AgroAsiaSiev, but not significantly different from aggressiveness of European isolates, regardless of their host of origin (*M*. × *domestica* or *M. sylvestris*). The response of isolates from *M*. × *domestica* was different across populations. Isolates from *M*. × *domestica* collected in Asia (AgroAsiaDom) were significantly more aggressive than isolates from Europe (AgroEuDom) on *M. sieversii*. The lower aggressiveness and the lower frequency of virulent isolates in European population from the agro-ecosystem support the view that, unlike in Central Asia, pathogen populations in Europe evolved a quantitative specialization to the apple-based agro-ecosystem.

For tests on *M*. × *domestica* accessions, the best model included the factor ‘*Malus* species of origin’ (BIC = 3709), significantly improving the null model (BIC = 3715; *P* < 0.001). There was no significant effect of the cultivar, cv. Gala and cv. Top Red having similar responses. Thus, adding this trait to the model did not significantly improve the BIC score (BIC = 3721). The most aggressive isolates were those from *M*. × *domestica* regardless of their geographic origin (Asia or Europe) (Fig. 2). However, isolates from *M*. × *domestica* were not significantly more aggressive than isolates from AgroAsiaSiev (*P* = 0.81). WildEuSylv was the least aggressive population, suggesting that the emergence of the disease on *M. sylvestris* was followed by the evolution of quantitative specialization in pathogen populations.

The number of isolates virulent on *M. sylvestris* accessions was low (Table 2). Furthermore, the severity of the disease caused by virulent isolates was weak (Fig. 2). As a consequence, the statistical analysis of aggressiveness had too low a power to infer any reliable conclusion and is therefore not presented.

## Discussion

Previous studies exploiting population genetic inference revealed a marked impact of domestication on fungal pathogen population structure, leading in some cases to the emergence of novel pathogen species (Couch et al. 2005; Munkacsi et al. 2007; Stukenbrock et al. 2007). However, the impact of domestication on pathogenicity traits has rarely been investigated, despite being of major importance to understand the consequences of modern human activities on disease emergence. We used cross-pathogenicity tests in controlled quarantine conditions to investigate the changes in pathogenicity traits of the apple scab fungus, *V. inaequalis*, associated with the domestication and spread of its host. Our main findings were that host-tracking was associated with a change in virulence and an increase in aggressiveness of pathogen populations from the agro-ecosystem. Our results suggested that the transition from wild to apple-based agro-ecosystem did not promote the evolution of specialized populations of *V. inaequalis*, as they were still able to infect the ancestral host plant. In contrast, host-range expansion from the domesticated apple to the European wild apple was associated with a certain degree of host specialization, as populations of *V. inaequalis* from *M. sylvestris* caused a less severe disease on *M.* × *domestica*.

### Changes in pathogenicity traits associated with host-tracking

The pathogenicity of isolates collected in natural ecosystems on the wild apple *M. sieversii* (WildAsiaSiev) can be compared to more recently founded pathogen populations from the agro-ecosystem to draw inferences on the evolutionary changes associated with the emergence and spread of *V. inaequalis* on the domesticated apple. The lack of pathogenicity of isolates from the WildAsiaSiev population when inoculated onto the domesticated apple suggests a strong ecological differentiation between the WildAsiaSiev population and the populations from the agro-ecosystem. A similar pattern of pathogenicity was observed in a study on the rice blast fungus where isolates of *M. oryzae* from ancestral undomesticated hosts (Setaria millet) were not able to infect or were less aggressive on domesticated rice (Couch et al. 2005). Unlike *M. oryzae*, however, *V. inaequalis* did not emerge on the domesticated crop following a host-shift but through a more continuous process of host-tracking. It could be hypothesized that the disruptive change during domestication corresponded to input of new resistance genes in the domesticated apple species. Indeed, along the process of domestication, *M*. × *domestica* might have hybridized with several wild species of *Malus* such as *M. sieversii*, *M. baccata*, *M. kirghisorumi, M. orientalis* (Coart et al. 2006; Forsline et al. 2010; A Cornille, P. Gladieux, I. Roldán-Ruiz, F. Laurens, B. Le Cam, M. J. M. Smulders, A. Nersesyan, J. Clavel, M. Olonova, L. Feugey, I. Gabrielyan, X. G. Zhang, M. I. Tenaillon, T. Giraud, unpublished manuscript) from which new resistant genes could have been introgressed. Resistance genes from *M. sieversii* might also be unconsciously selected during the course of domestication. A recent study also suggests that novel resistant alleles may also have been created in crops during domestication (Zhai et al. 2011).

Additional insights into the changes in pathogenicity associated with the apple domestication can be gained by comparing the frequency of virulence and aggressiveness of isolates collected in populations from natural and agro-ecosystems. The different populations could only be compared on *M. sieversii*, as isolates from the WildAsiaSiev population were avirulent on *M.* × *domestica*. Isolates from the WildAsiaSiev population were less aggressive and less frequently virulent on *M. sieversii* than isolates from the population representing the Central Asian agro-ecosystem (AgroAsiaSiev and AgroAsiaDom populations), suggesting that spread of the disease on the domesticated apple may have been associated with a quantitative increase in pathogenicity. These differences in overall pathogenicity (both virulence and aggressiveness) may be related to the contrasted ecological properties of the two environments. Higher density and homogeneity of the agro-ecosystem would have promoted higher pathogenicity of populations infecting domesticated hosts, while the patchy and geographically structured populations of *M. sieversii* (Richards et al. 2009) would have impeded the evolution of high pathogenicity in populations from natural ecosystems. The lower overall pathogenicity of WildAsiaSiev could also be explained by differences in resistance to *V. inaequalis* between *M. sieversii* populations from Central Asian mountains and plains. A study of local adaptation using *M. sieversii* genotypes from which WildAsiaSiev and AgroAsiaSiev populations were sampled would be interesting for assessing to what extent WildAsiaSiev and AgroAsiaSiev populations are adapted to their host, taking into account the potential diversity in resistance to *V. inaequalis* existing within *M. sieversii.*

### Apple-based agro-ecosystem did not promote the evolution of specialized populations of *V. inaequalis*

The higher level of environmental homogeneity of the agro-ecosystem is thought to promote the ecological specialization of pathogens associated with cultivated species (Stukenbrock and McDonald 2008). Following host-tracking*, V. inaequalis* populations on *M*. × *domestica* did not loose their capacity to infect their wild native host *M. sieversii*. In Central Asia, specialization of the *V. inaequalis* population from *M*. × *domestica* agro-ecosystem might have been impeded by recurrent gene flow between populations infecting domesticated apples and neighbouring populations from *M. sieversii* in human-managed habitats. In Europe, pathogenicity experiments supported quantitative specialization of the *V. inaequalis* population from the agro-ecosystem. This population was less frequently virulent and less aggressive than Asian populations from the agro-ecosystem on *M. sieversii*. Allelic combinations providing higher pathogenic fitness on *M. sieversii* would have been unnecessary in European populations and would thus have been progressively lost through genetic drift. The observed quantitative specialization in Europe could therefore be due to geographic distance rather than to environmental differences between wild and agricultural ecosystems.

### The emergence of apple scab on the European wild apple resulted from a host-range expansion associated with quantitative host specialization

The pathogenicity of isolates from European crabapple on the domesticated trees indicated that the emergence of apple scab on *M. sylvestris* resulted from a host-range expansion and not a host-shift. Analyses further revealed that the host-range expansion from *M.* × *domestica* to *M. sylvestris*, nevertheless, led to a quantitative specialization with a lower frequency of virulence and lower aggressiveness of populations from *M. sylvestris* on the native host *M*. × *domestica*, suggesting a trade-off between adaptations onto these two hosts. The lower phylogenetic distance between *M. sieversii* and *M.* × *domestica* (Velasco et al. 2010) compared with that between *M.* × *domestica* and *M. sylvestris* could explain the less pronounced trade-off between adaptations to these two former hosts. Stronger trade-offs are indeed expected for host-range expansion involving phylogenetically distant hosts than for host-tracking. Isolates from *M. sylvestris* were not more aggressive on *M. sylvestris* than isolates sampled on other hosts. The highly scattered distribution of *M. sylvestris* across Europe and within forests may not promote evolution towards higher aggressiveness (Gilbert 2002). Environmental context could thus be of primary importance for the evolution of pathogenicity and aggressiveness.

### Concluding remarks

We have investigated the changes in pathogenicity in populations of the pathogen *V. inaequalis*, associated with the domestication of their host. The emergence of *V. inaequalis* on domesticated hosts was associated with a gain in virulence and a subsequent increase in aggressiveness on *M. sieversii* trees in contact with the agro-ecosystem. These results are expected characteristics of the domestication syndrome in pathogens. We hypothesize that pathogen populations did not specialize on the domesticated host because of the close relatedness between their original wild host and *M.* × *domestica* and the persistence of gene flow between pathogen populations from *M. sieversii* and *M.* × *domestica* in Central Asia. A decrease in the levels of gene flow in Europe would have led to a decrease in aggressiveness of the European population from *M.* × *domestica* but without a loss of its capacity to infect its original wild host. The introduction of *V. inaequalis* in Europe was followed by a host-range expansion from *M.* × *domestica* to the more phylogenetically distant *M. sylvestris*. The existence of efficient resistance traits in this wild species, in association with its very low density in forests, may have limited the increase in aggressiveness of the corresponding pathogen population.

Our findings have important implications regarding the assessment of risk for the emergence of highly aggressive pathogens in wild and agricultural ecosystems. We show here that regulation agencies, policy makers, as well as plant breeders, should consider very carefully the risk of host-tracking by pathogens onto domesticated species. The favourable environment provided by the agro-ecosystem can foster the emergence of new pathogens with increased virulence and aggressiveness. While many plant and animal species are still under domestication (Diamond 2002), our results point to the considerable risk that potentially unnoticed pathogens can adapt via host-tracking and subsequently spread across continents. Moreover, the finding that a pathogen having emerged in the agro-ecosystem has increased its aggressiveness without loosing its ability to infect its original host also suggests that such pathogens can subsequently pose serious threats to wild crop relatives. The policy regarding cultivated areas should therefore take into account the surrounding wild ecosystem to prevent a ‘boomerang’ effect, that is, the return of more aggressive pathogens back on wild original hosts.

## Data archiving statement

Data deposited in the Dryad repository: doi: 10.5061/dryad.6bp470fn

## Supporting Information

**Figure S1.** Geographic location of strains (circles) and hosts (squares) sampling sites. Populations of strains are identified by different colours: WildAsiaSiev (red), AgroAsiaSiev (orange), AgroAsiaDom (purple), AgroEuDom (yellow) and WildEuSylv (green). Red squares represent the three accessions of *M. sieversii* from Kazakhstan. Accessions of *M. sylvestris* are located in France in the Rambouillet forest (next to Paris). Maps are provided by Google Earth©.

**Data S1.** *M. sylvestris* accessions were genotyped using microsatellite markers, and their assignment to the gene pool of their putative species of origin was checked using a reference data set.
