# Supplementary material for: Evolution of pathogenicity traits in the apple scab fungal pathogen in response to the domestication of its host
Source: Evol Appl. 2012 Nov;5(7):694–704. doi: 10.1111/j.1752-4571.2012.00246.x (PMC3492895; doi:10.1111/j.1752-4571.2012.00246.x)
Supplement: Supplementary file 1 [file eva0005-0694-SD1.doc]

**Supplementary materials S1**

Geographic location of strains (circles) and hosts (squares) sampling sites. Populations of strains are identified by different colours: WildAsiaSiev (red), AgroAsiaSiev (orange), AgroAsiaDom (purple), AgroEuDom (yellow) and WildEuSylv (green). Red squares represent the three accessions of *M. sieversii* from Kazakhstan. Accessions of *M. sylvestris* are located in France in the Rambouillet forest (next to Paris). Maps are provided by Google Earth©

**Supplementary materials S2**

*Malus sylvestris* can produce hybrids with *M.* × *domestica* in Europe that are difficult to identify based on morphological traits in the field. To examine the actual proportion of *M. sylvestris* ancestry in our samples of European wild apples, we used data from multilocus microsatellite typing to assign individuals to a reference individual from Gladieux *et al.* (2008). The DNA of plant specimens was extracted from frozen leaves using Macherey’s Nucleospin 96 Plant and genotyped using 13 microsatellite loci: Ch01f02, Ch01f03, Ch01h10, Ch01h01, Ch02c06, Ch02c09, Ch02c11, Ch02d08, Ch04c07, Ch04e05, Ch05f06, GD12 and Hi02c07 (Patocchi *et al.*, 2009). Multiplex microsatellite amplifications PCR was performed using the Multiplex PCR Kit (QIAGEN, Inc.) following the manufacturers protocol in a final reaction volume of 15 µL (7.5 µL of QIAGEN Multiplex Master Mix, 0.15 µL of primer mix with the forward primers labelled using a fluorescent dye, 4 μL of H2O and 2 μL of template DNA). Cycling conditions used for touch-down PCR started with a 15 min denaturation step at 95 °C followed by 5 cycles of 30 s for initial denaturation at 94 °C, 90 s of annealing at 60 °C with a reduction in temperature of 1 °C per cycles, 60 s of extension at 72 °C then 30 cycles of 30 s for initial denaturation at 94 °C, 90 s of annealing at 55 °C, 60 s of extension at 72 °C and 15 min of final elongation step at 72 °C. Genotyping was performed at INRA Clermont-Ferrand on an ABI PRISM 3100, using 0.2 μL of GS500(-250)LIZ size standard (Applied Biosystems). Allele scoring was carried out using the Genemapper 4.0 software (Applied Biosystems).

All analyses on this dataset were performed using the Bayesian clustering method implemented in the Structure 2.1 program (Pritchard *et al.*, 2000; Falush *et al.*, 2003). Structure runs consisted in a burn-in period of 30000 iterations followed by a run length of 300000 iterations, and a model with correlated allele frequencies and admixture among populations. Each run was repeated 10 times to check for convergence of the Monte Carlo Markov Chain (MCMC).

The microsatellite data set represents 59 individuals of the two *Malus* species: 44 *M.* × *domestica* as reference individuals, 15 *M. sylvestris* (11 reference individuals selected from *Gladieux et al.* (2008) and four analysed samples). Reference individuals were selected after running the program to identify the genotypes most representative of the *M. sylvestris* and *M.* × *domestica* gene pools. Individuals were assigned to two distinct clusters without using any prior information as to their origin. Individuals with membership proportion lower than 80% in their species’ population were removed to construct the data set of reference genotypes used in subsequent assignment analyses.

The four individuals originally labelled as *M. sylvestris* and from which *Venturia inaequalis* isolates were inoculated had an average membership proportion of 99% in the *M. sylvestris* reference cluster. This demonstrates that the wild European apple trees on which we have inoculated *V. inaequalis* are good representatives of the *M. sylvestris* gene pool.

**References**

Falush, D., M. Stephens, and J. K. Pritchard. 2003. Inference of population structure using multilocus genotype data: linked loci and correlated allele frequencies. Genetics 164:1567-1587.

Gladieux, P., X. G. Zhang, D. Afoufa-Bastien, R. M. V. Sanhueza, M. Sbaghi, and B. Le Cam. 2008. On the Origin and Spread of the Scab Disease of Apple: Out of Central Asia. Plos One 3, e1455.

Patocchi, A., A. Frei, J. E. Frey, and M. Kellerhals. 2009. Towards improvement of marker assisted selection of apple scab resistant cultivars: *Venturia inaequalis* virulence surveys and standardization of molecular marker alleles associated with resistance genes. Molecular Breeding 24:337-347.

Pritchard, J. K., M. Stephens, and P. Donnelly. 2000. Inference of population structure using multilocus genotype data. Genetics 155:945-959.
